# Effect of Surface Roughness of Deciduous and Permanent Tooth Enamel on Bacterial Adhesion

**DOI:** 10.3390/microorganisms10091701

**Published:** 2022-08-24

**Authors:** Bernardo Teutle-Coyotecatl, Rosalía Contreras-Bulnes, Laura Emma Rodríguez-Vilchis, Rogelio José Scougall-Vilchis, Ulises Velazquez-Enriquez, Argelia Almaguer-Flores, Jesús Angel Arenas-Alatorre

**Affiliations:** 1 Facultad de Odontología, Centro de Investigación y Estudios Avanzados en Odontología (CIEAO), Universidad Autónoma del Estado de México, Toluca, Estado de México C.P. 50130, Mexico; 2 Laboratorio de Biointerfases, Facultad de Odontología, Universidad Nacional Autónoma de México, Delegación Coyoacán, Ciudad de México C.P. 04510, Mexico; 3 Instituto de Física, Universidad Nacional Autónoma de México, Delegación Coyoacán, Ciudad de México C.P. 04510, Mexico

**Keywords:** enamel, deciduous, permanent, surface roughness, bacterial adhesion, caries

## Abstract

The adhesion of some bacteria has been attributed to critical levels of roughness in hard tissues, which increases the risk of developing caries. The objective of this work was to assess the effect of deciduous and permanent tooth enamel surface roughness on bacterial adhesion. One hundred and eight samples of deciduous and permanent enamel were divided into two groups (*n* = 54). G1_DE deciduous enamel and G2_PE permanent enamel. The surface roughness was measured by profilometry and atomic force microscopy (AFM). Subsequently, the evaluation of bacterial adherence was carried out in triplicate by means of the XTT cell viability test. Additionally, bacterial adhesion was observed using scanning electron microscopy (SEM) and confocal laser scanning microscopy (CLSM). The average values of the micrometric roughness in both groups were similar; however, in the nanometric scale they presented significant differences. Additionally, the G1_DE group showed the highest amount of adhered *S. mutans* and *S. sanguinis* compared to the G2_EP group. Although the roughness of deciduous and permanent enamel showed contrasting results according to the evaluation technique (area and scale of analysis), bacterial adhesion was greater in deciduous enamel; hence, enamel roughness may not be a determining factor in the bacterial adhesion phenomenon.

## 1. Introduction

Dental enamel is known to be the hardest tissue in the human body and is composed of 92–96% inorganic matter or mineral phase, 3% water, and 1% organic material by weight [1,2]. This tissue presents a complex hierarchical assembly that goes from the nanoscale to the microscale and possesses anisotropic properties [3,4]. In general, the topography of any surface will take the form of a series of peaks and valleys, which may vary in both height and spacing [5]. This can be characterized by a set of parameters (roughness) that give information about the height and distribution of the features on the surface, but it varies enormously according to the measurement scale chosen. Atomic force microscopy (AFM) and optical and stylus profilometers are methods commonly used to characterize it [6]. Specifically, in dental enamel, the topography is given by its microstructure, represented by hydroxyapatite crystals arranged in prisms or rods that create a wide variety of structures and morphologies [7,8]. The enamel surface of erupted and functional teeth may be irregular due to structural variations, which are a consequence of the occlusal function or the oral environment condition. In addition, deciduous enamel shows smoother surfaces than permanent enamel [9].

The effect of hard tissue surface roughness on microbial retention is a complex and controversial phenomenon. For a long time, the literature has stated that the roughness of intraoral hard surfaces has a great influence on the retention of oral microorganisms and that an increase in surface roughness results in a faster colonization of surfaces and a faster maturation of plaque, leading to an increased risk of caries [10]. Caries is a biofilm-mediated, sugar-driven, multifactorial, dynamic disease resulting in the phasic demineralization and remineralization of dental hard tissues [11]. To date, dental caries in patients with primary or mixed dentition is a major public health problem since it is still the most prevalent chronic disease. In this sense, the Global Burden of Untreated Caries reported that untreated caries in deciduous teeth was the 10th most prevalent condition, affecting 621 million people worldwide (9%); in addition, untreated caries in permanent teeth affected 2.4 billion people worldwide (35%) [12].

On the other hand, bacterial adhesion is the first step of biofilm formation; that phenomenon can be influenced by the surface roughness because it provides a greater surface area for bacterial adhesion and protects microorganisms from shear force [13,14]. The initial colonization process is dominated by oral streptococci [15], in which we find *Streptococcus sanguinis*, considered as an early colonizer, and non-mutans streptococci [16,17], as well as *Streptococcus mutans*, the principal pathogenic agent in caries [18] and early childhood caries [19] due to its ability to metabolize sucrose into lactic acid to synthesize acids in a low pH environment and produce extra- and intra-cellular polysaccharides [18]. Interspecies interaction of oral streptococci plays an important role in caries development [15].

Finally, the adhesion of some bacteria has been attributed to critical levels of roughness on hard tissues. The irregularities protect bacteria from shear forces and allow them to come into contact with the surface and form biofilms, thus increasing the risk of developing caries. The deciduous and permanent enamel present morphological differences that can affect their topography and the development of caries. The lack of studies that jointly describe the effect of surface roughness on bacterial adhesion creates the need to conduct novel studies on both tissues using similar techniques in order to elucidate the phenomenon. Thus, the purpose of this study was to assess the effect of deciduous and permanent enamel surface roughness on bacterial adhesion. To date, no similar investigation has been carried out. This study’s two null hypotheses were that there are differences in (a) surface roughness and (b) adhesion of some oral streptococci between deciduous and permanent enamel.

## 2. Materials and Methods

### 2.1. Tooth Selection and Sample Preparation

The present study is based on a previous assessment of deciduous (*n* = 54, G1_DE) and permanent (*n* = 54, G2_PE) dental enamel samples. Primary incisors and premolars without decay, fluorosis, dental fractures, or fillings and extracted from non-bruxed healthy young patients (aged 6 to 17 years) were collected under informed consent. The study was submitted to an Ethical Committee Board and approved (CEICIEAO-2021-019). The included teeth were cleaned and stored at 4 °C in 0.2% thymol solution prior to analysis. Subsequently, enamel fluorescence was evaluated using two devices (DIAGNOdent^®^ (DI-AGNOdent^®^, KaVo, Biberach, Germany) and The Spectra™ (Air Techniques, Melville, NY, USA)); only teeth with healthy enamel were included in the study. The crown of each tooth was separated from its root by means of a diamond disk (BesQual, New York, NY, USA) mounted on a low-speed motor (micromotor STRONG 207S-106, Saeshin Precision Co., Ltd., Daegu, Korea) under constant irrigation to avoid dehydration. Then, the crown was fixed to a glass slide with thermoplasticized epoxy resin (Allied High Tech Products, Rancho Dominguez, CA, USA). Subsequently, enamel blocks (3 × 3 × 1 mm) from the buccal and lingual surface were extracted with a diamond blade (South Bay Technology, Inc., San Clemente, CA, USA) mounted on a cutter (South Bay Technology, Inc., San Clemente, CA, USA) under irrigation. Finally, all samples were rinsed with deionized water and dried at room temperature for the baseline surface roughness evaluation [20,21].

### 2.2. Surface Roughness Analysis

Surface roughness of deciduous and permanent enamel was evaluated using two different scaling techniques.

### 2.3. Profilometry

The surface roughness of the enamel blocks was evaluated by a mechanical profilometer instrument (Mitutoyo Surftest SJ-301, Tokyo, Japan). Surface roughness values were obtained using the 5 µm radius diamond tip with a cut off value of 0.08 mm (λc), a transverse length of 0.5 mm, a speed of 0.25 mm/sec, and a Gaussian Filter. This procedure was performed on three different locations at the center of the samples [20,21]. For each sample, the mean values of the parameters Ra (the average distance from the profile to the mean line over the length of assessment) and Rz (the peak-to-valley values of five equal measures within the profile) were obtained under ISO 4287-1997 [22]

### 2.4. Atomic Force Microscopy

Three representative specimens randomly selected samples from each group were evaluated through an atomic force microscope (NaioAFM, Nanosurf, Liestal, Switzerland) using a previous methodology [23] with some modifications. A total of five representative images with a 50 × 50 μm area were acquired in tapping mode for each specimen at each location. All AFM images were processed with the Naio control software version 3.10.0 (Nanosurf, Liestal, Switzerland); the Ra and Rz parameters were determined in nanometers (nm) across independent scans areas per specimen and expressed in micrometers (µm) as mean ± standard deviation to compare these results with the ones obtained using the profilometry.

### 2.5. Bacterial Adhesion Test

The *S. mutans* ATCC 25,175 and *S. sanguinis* ATCC 10,556 strains used in this study were grown separately on Hemin–Vitamin K (HK)-enriched agar plates (brain/heart infusion agar (BBL; Becton, Dickinson, NJ, USA), trypticase soy agar (BBL; Becton, Dickinson), and yeast extract (BBL; Becton, Dickinson), supplemented with 5 µg/mL hemin (Sigma-Aldrich, St. Louis, MO, USA), 0.3 µg/mL menadione (Sigma-Aldrich), and 5% defibrinated sheep blood(Microlab, Mexico City, Mexico) at 37° C for 7 days, under anaerobic conditions (80% N_2_, 10% CO_2_ and 10% H_2_) [20].

Before carrying out the bacterial adhesion test, the enamel samples were washed for 10 min and sterilized at 120 °C for 15 min [20,21,24,25,26].

Bacterial growth from 7-day cultures of each strain was harvested and the optical density (OD) in each tube was adjusted at 1–600 nm in a spectrophotometer (Eppendorf BioPhotometer D30, Hamburg, Germany). Sterile enamel blocks were placed individually into a 96-well cell-culture plate with a flat bottom (Costar Cat# 3599, Corning, NY, USA). A total of 10^8^ cells/mL suspension of each reference strain was individually added, in a total volume of 200 µL. Plates were incubated for 24 h at 37° C under anaerobic conditions using Hemin–Vitamin K (HK)-enriched broth media (5 µg/mL hemin and 0.3 µg/mL menadione) to allow bacterial adhesion on the enamel surfaces. All the experiments were performed using three samples per bacterium and group.

After the anaerobic incubation time, each enamel sample was washed three times with Hemin–Vitamin K (HK)-enriched broth media to eliminate non-adherent bacteria and determine the number of adhered bacteria by means of the XTT cell viability assay [20,21].

### 2.6. XTT Cell Viability Assay

Bacterial adhesion on dental enamel was evaluated using XTT Cell viability kit assay (Cell Signaling Technology^®^ #9095, Danvers, MA, USA); 50 µL of the XTT reagent, and 100 µL of the Hemin–Vitamin K (HK)-enriched broth media were added to each well and incubated under anaerobic conditions in the dark for 4 h at 37 °C. After incubation, 100 µL of all solutions were transferred onto a new sterile microtiter plate and analyzed at 450 nm (reference wavelength, 620 nm) using the multi-mode microplate reader FilterMax™ F5 (Molecular Devices, San Jose, CA, USA). Finally, the number of cells/mL was obtained with the transformation of absorbance values through a standard calibration curve [20,21].

### 2.7. Confocal Laser Scanning Microscopy

LIVE/DEAD^®^ BacLight™ Bacterial Viability Kits (Invitrogen Ltd., Paisley, UK) [20,21] were used for microscopical analysis of bacterial adhesion. After anaerobic incubation, one sample per group was gently removed from a 96-well plate, rinsed, and stained with LIVE/DEAD^®^ BacLight™ [20]. The stained bacteria were evaluated in the confocal laser scanning microscopy (CLSM) Olympus Fluoview FV 1000 Spectroscopic Confocal System (CLSM FV 1000 Olympus, Tokyo, Japan) with a UPLSAPO 100× O NA: 1.4 lens (Olympus, Tokyo, Japan) immediately after the staining procedures [21].

### 2.8. Scanning Electron Microscopy

After anaerobic incubation, specimens (one per group) were fixed in 2.0% glutaraldehyde for 24 h at room temperature; afterwards, they were washed with phosphate-buffered solution (pH 7.4) and dehydrated in a graded series of ethanol (20 to 100%). Then, samples were vacuum dried and sputter-coated with gold. Finally, samples were examined in an SEM JEOL (JEOL-JSM-6610LV, JEOL USA, Inc., Peabody, MA, USA) at 20 kV [20,21] in order to obtain high-resolution images of the bacterial cells.

### 2.9. Statistical Analysis

Data were analyzed by SPSS (Statistical Package for Social Science) program, version 24 (SPSS IBM., New York, NY, USA). Through a Kolmogorov–Smirnov test, the data distribution was evaluated, and the comparisons of roughness values (Ra and Rz) between two groups were performed by pairwise Mann–Whitney U test. Additionally, the number of bacteria of the same species that adhered to deciduous and permanent enamel were determined by the pairwise Mann–Whitney U test; *p* < 0.05 was considered to be statistically significant.

## 3. Results

### 3.1. Surface Roughness

The average values for the roughness parameters Ra and Rz by each characterization method are shown in Table 1.

*Profilometry*: the group G1_DE showed the minimum values of surface roughness with no statistically significant differences (*p* > 0.05).

AFM: the group G2_PE exhibited the lowest values of surfaces roughness with significant differences (*p* < 0.05).

**Figure 1:**
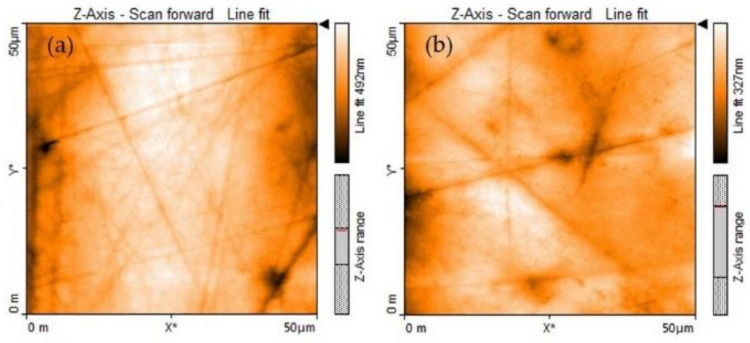
AFM images from dental enamel; (**a**) deciduous, (**b**) permanent. The asterisk denotes the image axes (i.e., X*, Y*).

Representative AFM images are shown in Figure 1. They reflect the surface topographies of the enamel of deciduous and permanent enamel in a 50 × 50 µm area by AFM. The surface of the deciduous enamel showed less marked microporosities, wear grooves and homogeneous scratches in comparison to the permanent enamel.

### 3.2. XTT Cell Viability Assay

The number of bacterial cells attached to the enamel samples is shown in Table 2. The G1_DE group showed the highest amount of *S. mutans* and *S. sanguinis* adhered compared to the G2_PE group.

### 3.3. CLSM Observations

**Figure 2:**
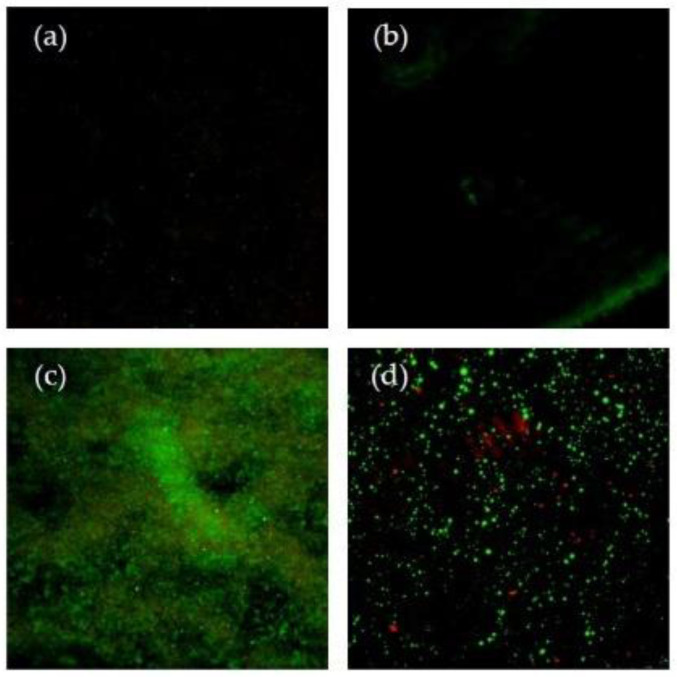
Confocal laser scanning microscopic images (xy-views) of the bacterial cells that adhered to dental enamel assayed by LIVE/DEAD^®^ BacLight™ Bacterial Viability Kits (magnification 100×); (**a**) deciduous enamel with *S. mutans*; (**b**) permanent enamel with *S. mutans*; (**c**) deciduous enamel with *S. sanguinis*; (**d**) permanent enamel with *S. sanguinis*.

Typical live/dead confocal images of biofilm staining formed on deciduous and permanent enamel surfaces are presented in Figure 2. The image series confirms, qualitatively, the differences in bacterial adhesion determined by the XTT cell viability assay. The deciduous enamel presented greater amounts of adhered bacteria (*S. mutans* and *S. sanguinis*) than permanent enamel; in addition, *S. sanguinis* was the bacterium that had more adherence to both substrates. The live cells can be seen in green color; they were scattered in all the substrates tested.

### 3.4. SEM Observations

**Figure 3:**
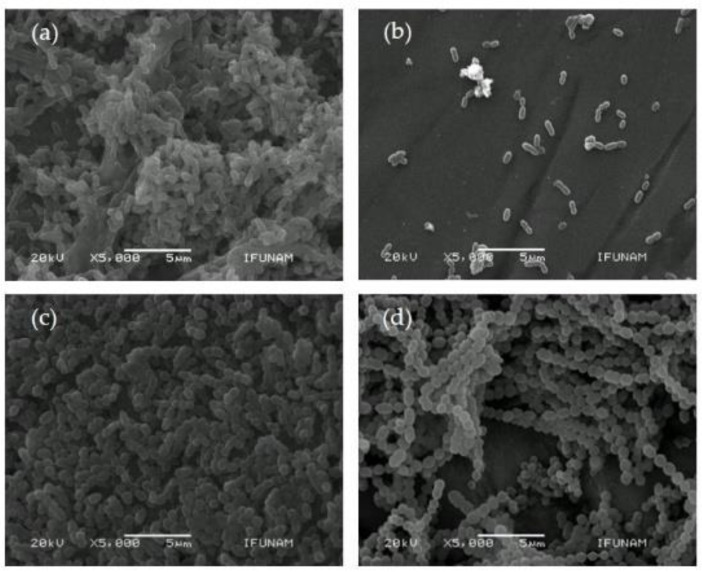
Representative scanning electronic microscopic (SEM) of dental enamel incubated with microorganisms; original magnification, 5000×; scale bar = 5 µm; (**a**) deciduous enamel with *S. mutans*; (**b**) permanent enamel with *S. mutans*; (**c**) deciduous enamel with *S. sanguinis*; (**d**) permanent enamel with *S. sanguinis*.

Figure 3 shows representative micrographs of *S. mutans* and *S. sanguinis* that adhered to the deciduous and permanent enamel. At 5000× magnification, the deciduous and permanent enamel surfaces can be seen with a large amount of disseminated oral streptococci covering them. The micrographs qualitatively validate the results from the XTT cell viability assay.

## 4. Discussion

The surface roughness of deciduous and permanent enamel was evaluated using both a profilometer and an AFM. The results yielded by the two different devices were presented and compared. Additionally, the adhesion of oral streptococci was also evaluated.

The enamel samples were not grounded or mechanically treated to keep the ultrastructure of both substrates intact. Therefore, we could observe the influence of the surface per se on bacterial adhesion, since permanent and deciduous human teeth have a perfectly enamel smooth surface [27]. SEM and reflectance confocal microscopy (RCM) images of deciduous and permanent enamel showed smooth surfaces with little areas of irregularities, furrows, and unevenness of variable depth [9,27]. In addition, SEM micrographs of caries-free bicuspid teeth have shown areas of closed and exposed prisms as well as natural fractures [28]; those of the primary mandibular central incisors exhibit smooth surfaces with some grooves [29].

### 4.1. Surface Roughness

In order to carry out an exhaustive evaluation of the surface roughness of deciduous and permanent enamel at different scales, two instruments were used. On one hand, the profilometer was selected since it is an instrument that has a large dimensional scale capable of measuring rough surfaces [30]. On the other hand, the AFM was used, because it is a suitable microscopic method to analyze biological objects at nanoscale under natural conditions (no exhaustive preparation) [31].

The results obtained by profilometry showed that both deciduous and permanent enamel presented similar roughness, which is why the first null hypothesis was rejected, despite deciduous enamel presenting slightly lower roughness values (−19%). The low surface roughness observed on deciduous enamel may be due to the presence of a prismless enamel that covers all healthy deciduous teeth [32,33,34].

Lucchese et al. [9] illustrated a method of SEM digital image processing that is able to quantify and discriminate the morphological characteristics of deciduous and permanent tooth enamel by means of a digital processed algorithm, such as the roughness index (RI). They found that permanent enamel surface had RI values higher than deciduous enamel. As can be observed, their results differ from ours; this can be attributed to the use of a different surface roughness evaluation method.

Regarding the outcomes derived from the AFM height scans, these results showed that deciduous tooth enamel significantly doubles the roughness values of permanent enamel. Some chemical and histological differences between primary and permanent teeth, such as lower mineralization, higher porosity, and high organic content of primary teeth [35,36], makes the enamel less hard [37] and more susceptible to demineralization and wear. Furthermore, according to some authors, tooth brushing damages deciduous enamel [38,39]. All the reasons stated above could be the cause of deciduous enamel presenting greater nanoscale roughness. In addition, it should be noted that deciduous teeth have spent their entire useful life cycle in the mouth under certain deleterious conditions that could accentuate their surface roughness, while the included permanent teeth in this study have been extracted for orthodontic reasons in young patients.

The results obtained through nanoprofilometry by AFM must be interpreted rationally and cannot be generalized since they were taken from micrometric areas and can increase proportionally when the size of the area to be evaluated increases [40]. Furthermore, comparisons of the surface roughness values of deciduous and permanent enamel obtained with the profilometer showed no statistically significant difference. However, significant differences were detected using the AFM. It is known that the different surface roughness values obtained from the same specimens may be a consequence of differences between the measurement sensitivity and operating mechanisms of the instruments employed (profilometer and AFM) [41]. On the other hand, the surface texture is a complex variable, and it is relatively simple to understand why many comparisons give highly variable results with the same surface [42].

To date, there are not enough studies that coincide with the results found because there are different techniques used to measure the surface roughness of the deciduous and permanent enamel. In addition, the conflicting data reported in the few available studies may be due to factors such as the variations between individual teeth, type, age, and ethnicity, region of tooth collection, the number of teeth analyzed, and the methodology employed by the researchers [2].

Regarding the qualitative evaluation of the deciduous and permanent enamel via the AFM images, it was observed that both substrates presented a typical morphology of healthy enamel as described in the literature [31,43]. Additionally, the deciduous enamel surface exhibited a greater amount of shallow or deep grooves of variable amplitude and irregular direction in comparison with permanent enamel. Those grooves have been reported by Hegedüs et al. [31] as sketches. The presence of grooves in both tissues has been attributed to the abrasion process present in erupted teeth due to tooth-brushing, diet, or certain habits [44], as well as the progressive changes that a tooth undergoes during life.

### 4.2. XTT Cell-Viability Assay

The direct evaluation of bacterial adhesion to the substrates studied was carried out using an XTT cell-viability assay, which is a colorimetric method for measuring metabolic activity and vitality through the reduction of the yellow salt XTT to a colored formazan product by dehydrogenases of metabolically active cells. Moreover, early colonizers (*S. sanguinis*) of the oral cavity were used in this task, since they play a key role in the adhesion process by adhering directly to the surface, facilitating the adhesion of the later colonizer (*S. mutans*).

The results obtained from the XTT cell viability test showed a marked adhesion of both streptococcal strains in the deciduous enamel compared to the permanent enamel under the same growth conditions. Probably the high content of organic material and the presence of abundant micro-porosities in the deciduous enamel [27,44] are the cause of this phenomena. Additionally, other factors like hydrophobicity and charge of dental enamel have a significant influence on the extent of bacterial adhesion [45]. In this study, the bacterial adhesion in both types of dental enamel was different; therefore, the second hypothesis was accepted.

Greater adhesion of *S. sanguinis* was found on both surfaces evaluated, despite not having been inoculated together. It has been reported that *S. sanguinis* (initial colonizer) presents a greater adhesion force to dental enamel in comparison with *S. mutans* (cariogenic bacteria) [46]. Moreover, *S. sanguinis* is surrounded by fibers with hair-like structures or clusters of fibrous hair [47] and possesses sialic acid lectins that can facilitate binding to hydroxyapatite [48]; such characteristics could contribute to the high adhesion of this microorganism to dental enamel.

The high affinity of *S. sanguinis* to both deciduous and permanent tooth enamel may be beneficial because both microorganisms have an antagonistic relationship [49] *S. sanguinis* uses H_2_O_2_ to compete with *S. mutans*, and bacteriocin of *S. mutans* can inhibit *S. sanguinis*; for this reason, when there are high quantities of *S. sanguinis* in the mouth, the colonization of *S. mutans* is avoided. Thus, high amounts of *S. sanguinis* have also been found in caries-free children [50,51]; hence, *S. sanguinis* could play a role as a “designer bacteria” that reduces the cariogenicity of the biofilms on enamel surface [23].

Despite *S. mutans* adherence in minor amounts to deciduous and permanent enamel, the values obtained in this research work exceed by thousands of percent the amount of mutans streptococci established as a caries risk in children (10^6^ colony-forming units per milliliter (CFU/mL)) [16]. Although the CFU/mL of saliva is not comparable with the CFU/mg of dental plaque or adhered cells/mL on the enamel, the caries risk values serve as a reference parameter to monitor the number of bacteria attached to the substrate studied.

As observed, deciduous teeth are more vulnerable to caries because they can be more colonized by *S. mutans*. Furthermore, they present differences in their chemical composition and their physical properties, including thinner, softer enamel that is more vulnerable to dissolution by cariogenic acids [52], Therefore, greater emphasis should be placed on establishing preventive strategies therapies at a very early age. The establishment of good oral hygiene habits, as well as the effective use of fluoridated pastes or topical fluoride, could minimize caries risk. Fluorine intervenes in the metabolism of the bacteria present in the biofilm and makes tooth enamel more resistant to acid attack.

### 4.3. CLSM Observations

According to the CLSM findings, deciduous enamel had highest amounts of live bacteria scattered throughout its surface. Live bacteria are seen in green color due to the penetration of the small molecule STYO 9 present in the LIVE/DEAD^®^ stain, which is capable of penetrating vital cells [53]. The dye mentioned above has been used to observed oral streptococci on deciduous and permanent enamel [20,21].

### 4.4. SEM Observations

As expected, in accordance with the XTT cell-viability assay results, SEM images showed a higher adhesion of bacteria on deciduous enamel than permanent enamel. This means that deciduous enamel is more prone to be colonized by the microorganisms studied. Specifically, *S.mutans* was observed in the bacillary way and as a single, double, or triple cell, without forming its classic chains. It is well-known that the bacillary way is due to its cell-division phase, its original morphology [54], and the acidity of its culture medium [55]; on the other hand, the absence of its classic morphology is a consequence of the anaerobic conditions in which it is cultured [56].

On one hand, it seems that the micrometric surfaces roughness of deciduous and permanent enamel is not an influential factor for bacterial adhesion, because the micrometric roughness values in deciduous enamel are similar to those found in permanent enamel; therefore, the chemical composition and other physical properties of deciduous and permanent tooth enamel could play a prominent role in bacterial adhesion. On the other hand, it seems that the nanoscale roughness of the deciduous enamel did influence the adhesion of streptococci, since there was a statistical difference between the nanoscale roughness of the deciduous enamel versus the permanent one. The available literature suggests that nanoscale surface roughness and topography may exert a greater influence on bacterial adhesion [5,57]. Further, it has been found that enamel microscale morphology may significantly alter the direct adhesion forces of bacteria [58].

Finally, according to an exhaustive review of the relevant scientific literature, there are no previous studies that have investigated the effect of roughness surface of intact deciduous and permanent enamel on bacterial adhesion, which precludes possibility of comparison and therefore discussion with similar studies; although this is a limitation of the study, at the same time, it opens a door in the field of bacterial adhesion on the surfaces of intact human dental enamel. Additional limitations are related to the in vitro design, which cannot perfectly simulate the conditions found in the oral cavity, and the small size of the sample due to the complexity, time spent, and costs of the methods employed for the roughness and microbiological analysis.

Further AFM studies could deepen the analysis of the surface roughness in deciduous and permanent dental enamel using more roughness parameters as well as evaluation of bacterial adhesion when wild-type strains of streptococci are grown. The use of fresh bacterial strains in microbiological tests would determine if the bacteria preserve the ability to adhere to intact enamel tooth.

## 5. Conclusions

Bacterial adhesion was greater in deciduous enamel; hence, enamel roughness may not be a determining factor in the bacterial adhesion phenomenon.

## Figures and Tables

**Table 1:** Means and standard deviations of roughness parameters Ra and Rz (µm) of deciduous and permanent enamel obtained by profilometer and AFM.

	Groups	Roughness Parameters							
		*Ra*				*Rz*			
***Profilometer*** **(*n* = 54 p/g)** **AFM** **(*n* = 3 p/g)**	G1_DE	0.210	±	0.110	a	1.840	±	1.120	a
	G2_PE	0.250	±	0.200	a	2.070	±	1.530	a
	G1_DE	0.067	±	0.030	a	0.082	±	0.038	a
	G2_PE	0.030	±	0.018	b	0.037	±	0.023	b

Groups with different letters in column are significantly different by characterization method; pairwise Mann–Whitney U test, *p* < 0.05.

**Table 2:** Average and standard error of bacterial cells of *S. mutans* and *S. sanguinis* that adhered to deciduous and permanent enamel obtained by XTT cell viability assay.

Groups (*n* = 3)	Number of Bacterial Cells × 10^6^/mL							
	*S. Mutans*				*S. Sanguinis*			
**G1_DE**	227.2	±	12.3	A, a	374.4	±	6.8	A, b
**G2_PE**	31.4	±	12.3	B, a	87.9	±	8.8	B, b

Capital letters in a column are comparisons of same bacteria between different group. Lower-case letters in a row are comparisons between bacteria in the same group. Letters being the same indicates lack of statistical difference (pairwise Mann–Whitney U test, *p* < 0.05).

## Data Availability

Not applicable.
